# Integrated OMICS guided engineering of biofuel butanol-tolerance in photosynthetic *Synechocystis* sp. PCC 6803

**DOI:** 10.1186/1754-6834-6-106

**Published:** 2013-07-25

**Authors:** Hongji Zhu, Xiaoyue Ren, Jiangxin Wang, Zhongdi Song, Mengliang Shi, Jianjun Qiao, Xiaoxu Tian, Jie Liu, Lei Chen, Weiwen Zhang

**Affiliations:** 1 Laboratory of Synthetic Microbiology, School of Chemical Engineering & Technology, Tianjin University, Tianjin 300072, P.R. China; 2 Key Laboratory of Systems Bioengineering, Ministry of Education, Tianjin 300072, P.R. China

**Keywords:** Butanol, Tolerance, Transcriptomics, Metabolomics, *Synechocystis*

## Abstract

**Background:**

Photosynthetic cyanobacteria have been recently proposed as a ‘microbial factory’ to produce butanol due to their capability to utilize solar energy and CO_2_ as the sole energy and carbon sources, respectively. However, to improve the productivity, one key issue needed to be addressed is the low tolerance of the photosynthetic hosts to butanol.

**Results:**

In this study, we first applied a quantitative transcriptomics approach with a next-generation RNA sequencing technology to identify gene targets relevant to butanol tolerance in a model cyanobacterium *Synechocystis* sp. PCC 6803. The results showed that 278 genes were induced by the butanol exposure at all three sampling points through the growth time course. Genes encoding heat-shock proteins, oxidative stress related proteins, transporters and proteins involved in common stress responses, were induced by butanol exposure. We then applied GC-MS based metabolomics analysis to determine the metabolic changes associated with the butanol exposure. The results showed that 46 out of 73 chemically classified metabolites were differentially regulated by butanol treatment. Notably, 3-phosphoglycerate, glycine, serine and urea related to general stress responses were elevated in butanol-treated cells. To validate the potential targets, we constructed gene knockout mutants for three selected gene targets. The comparative phenotypic analysis confirmed that these genes were involved in the butanol tolerance.

**Conclusion:**

The integrated OMICS analysis provided a comprehensive view of the complicated molecular mechanisms employed by *Synechocystis* sp. PCC 6803 against butanol stress, and allowed identification of a series of potential gene candidates for tolerance engineering in cyanobacterium *Synechocystis* sp. PCC 6803.

## Background

Due to its high energy content and superior chemical properties such as low volatility and corrosiveness, and its compatibility with the existing fuel storage and distribution infrastructure, butanol has been proposed as a good candidate for next-generation transportation biofuel [1,2]. Traditionally, bio-butanol can be produced by anaerobic Gram-positive bacteria, such as *Clostridium acetobutylicum* through a so-called acetone-butanol-ethanol (ABE) fermentation process [3,4]. Although significant improvements have been made in the past decades to increase efficiency of the ABE process through a combination of strain screening, genetic engineering and process optimization [5-8], butanol production from the fermentation processes is still not competitive economically. As one of the alternatives, photosynthetic cyanobacteria have recently attracted significant attention as a ‘microbial factory’ to produce biofuels and chemicals due to their capability to utilize solar energy and CO_2_ as the sole energy and carbon sources, respectively [9,10]. Recent synthetic biology efforts have led to successful production of *n*-butanol, isobutyraldehyde and isobutanol in cyanobacterium *Synechococcus elongatus* PCC 7942 [11,12], demonstrating the potentials of using engineered photosynthetic microbes for large-scale production of butanol or other biofuel products in the future.

Currently, the butanol production by the synthetic cyanbacterial systems is at a level of a few dozen or hundred milligrams per liter [11], much lower than the native *Clostridium* or even synthetic *Escherichia coli* systems [13-15]. To improve productivity, one of the key issues needed to be addressed is the low tolerance of the photosynthetic hosts to butanol [16,17]. The tolerance mechanism of native *Clostridium* strains to butanol has been well-studied [16-19]. For example, analysis of butanol tolerant transposon-insertion mutants of *Clostridium beijerinckii* NCIMB 8052 have led to the discovery that butanol-tolerance is associated with reduced activity of the enzyme, glycerol dehydrogenase [20]. Recently a functionally unknown protein (encoded by SMB_G1518) with a hypothetical alcohol interacting domain was also found negatively related to butanol tolerance [21]. In *E. coli*, a global transcription factor cyclic AMP receptor protein (CRP) was also engineered for increasing butanol tolerance [22]. However, currently information related to biofuel tolerance in cyanobacteria is very limited.

Recently various genome-wide approaches, such as genomic library enrichment and whole-genome sequencing of tolerant mutants were also employed to identify genes conferring enhanced tolerance to *n*-butanol in *E. coli*[23,24]. The results showed that microbes tend to employ multiple and synergistic resistance mechanisms in dealing with a single stress [17], and to fully interpret the complicated and synergistic tolerance mechanism, genome-wide based analytical approaches are necessary [25]. In a previous study, we investigated responses of *Synechocystis* sp. PCC 6803 (hereafter *Synechocystis*) to butanol using an iTRAQ - LC-MS/MS based proteomics, the results identified 303 proteins differentially regulated by butanol [26]. To further decipher responses at transcript and metabolite levels, and to identify gene targets relevant to butanol tolerance, in this study, we applied an integrated approach coupling quantitative RNA-seq transcriptomics approach, quantitative reverse-transcript PCR (qRT-PCR) and GC-MS based metabolomics to analyze cellular responses of *Synechocystis* to butanol exposure. The transcriptomic result revealed very similar response patterns as those identified by the previous proteomic analysis that multiple resistance mechanisms may be utilized in coping with butanol stress in *Synechocystis*[26]; and the metabolomic analysis showed that 46 chemically classified metabolites were differentially regulated by butanol treatment, including 3-phosphoglycerate, glycine and urea which were elevated in butanol-treated cells. The integrated analysis led to the identification of a series of potential gene targets and pathways for tolerance engineering, we then constructed gene knockout mutants for three selected butanol-induced genes, *sll0690*, *slr0947* and *slr1295*, and comparative phenotype analyses showed that their disruptions led to increased sensitivity to butanol, suggesting the gene targets identified can be used for engineering butanol tolerance in *Synechocystis*.

## Results and discussion

### Overview of RNA-Seq transcriptomics analysis

To make the transcriptomics data comparable with previous proteomics data, we used the identical sampling conditions for transcriptomics as our previous proteomic analysis [26]. As described previously, *Synechocystis* was grown in BG11 supplemented with 0.20% (*v*/*v*) butanol and cell samples of both control and butanol treatment were collected by centrifugation (8,000 × *g* for 10 min at 4°C) at 24 h, 48 h and 72 h, corresponded to middle-exponential, exponential-stationary transition and stationary phases of the cell growth, respectively.

**Figure 1:**
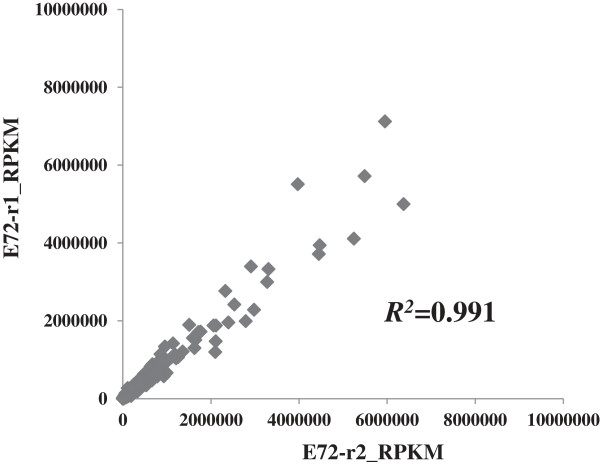
**Reproducibility of RNA-seq transcriptomic analysis.** Two biological replicates of butanol-treated samples were plotted. Normalized expression RPKM values were used. Correlation coefficient is indicated inside.

A total of 79.5-million raw sequencing reads was obtained from the RNA-seq transcriptomics analysis of six samples, with average reads of 13.2-million reads. After a two-step standard data filtering process, first to eliminate reads with low-quality bases (such as multiple N) and reads shorter than 20 bp, and then to eliminate sequence reads mapped to non-coding RNA of *Synechocystis*, a total of 27.5-million qualified mRNA-based sequence reads were identified (Table 1). The qualified sequence reads have an average genome mapping ratio of 66.4%. To assess the analytical reproducibility between biological replicates, we collected two biological replicates for butanol treated samples at 72 h, and plotted them using the normalized Reads Per Kilobase of Gene per Million Mapped Reads (RPKM) values, the result showed a correlation coefficient around 0.991 (Figure 1), indicating the overall good quality of RNA-sequencing based transcriptomics technology. The sequence reads matched to all 3189 coding genes in *Synechocystis* genome (data not shown), suggesting excellent sequencing depth and overall transcript coverage.

**Table 1:** Statistics of RNA-Seq transcriptomics analysis

**Sample ID**	**Raw sequence reads**	**Qualified mRNA reads**	**Genome mapped reads**	**Mapping ratio**
C242	15, 535, 317	5, 552, 572	3, 177, 168	57.22%
C483	9, 013, 667	2,331,738	1, 784, 966	76.55%
C722	8,986, 367	3, 026, 360	2, 455, 571	81.14%
B241	9, 175, 893	2, 947, 480	1, 755, 209	59.55%
B483	13,434,402	4,733,896	3,005,487	63.49%
B724	23, 330, 644	8, 945, 794	5, 430, 235	60.70%

Using a strict criterion of 1.5-fold change at all three time points (*i.e.*, 24, 48 and 72 h), we determined that 278 genes were induced upon butanol exposure, out of which 70 important genes with known functional categories were listed in Table 2. Functional category analysis of the induced genes showed that the most affected functional categories were “hypothetical proteins”, representing a total of more than 40% of all the up-regulated genes, consistent with the fact that nearly half of the genes in the *Synechocystis* genome are still annotated as hypothetical up to now [27]. Based on their expression level and regulation patterns by butanol, a subset of 10 genes was randomly selected for quantitative RT-PCR validation. Comparative RT-PCR analysis was performed for the genes between the butanol-treated sample and control at 48 h. The results showed very similar trends between qRT-PCR and RNA-Seq transcriptomics data (Table 3), suggesting a good quality of RNA-seq data.

**Table 2:** Important gene tragets induced by butanol

**Gene ID**	**Butanol *vs. *Control at 24 h**	**Butanol *vs. *Control at 48 h**	**Butanol *vs. *Control at 72 h**	**Description**
***Cell envelope***				
*sll0034*	2.52	1.54	1.86	D, D-carboxypeptidase
*sll0286*	2.33	1.90	1.55	Uncharacterized N-acetyltransferase
*sll0886*	1.88	1.78	2.79	UDP-N-acetylglucosamme-peptide N-acetylglucosammyltransferase-
*sll1053*	3.52	4.00	12.12	Membrane fusion protein *mtrc* precursor, putative
*sll1395*	1.91	2.61	1.54	dTDP-6--6-deoxy-L-mannose-dehydrogenase
*sll2010*	1.60	1.78	2.89	UDP-N-acetylmuramoylalanine--D-glutamate ligase
*slr0528*	2.52	1.91	2.45	UDP-N-acetylmuramoylalanyl-D-glutamate--2, 6-diaminopimelate ligase
*slr0993*	1.75	2.33	4.21	Putative peptidase
*slr1166*	2.86	1.68	2.36	UDP-glucose:tetrahydrobiopterin glucosyltransferase
*slr1196*	4.07	1.91	2.74	Periplasmic protein, function unknown
*slr1744*	1.89	1.92	2.42	N-acetylmuramoyl-L-alanine amidase, periplasmic protein
*slr2015*	3.56	4.15	2.39	Type 4 pilin-like protein, essential for motility
*slr2016*	4.40	1.71	1.71	Type 4 pilin-like protein, essential for motility
***Central intermediary metabolism***				
*sll0646*	4.03	1.87	1.96	Guanylyl cyclase
*slr0288*	1.65	2.28	2.58	Glutamate--ammonia ligase
*slr0899*	2.19	2.75	2.50	Cyanate lyase
*slr0940*	3.33	1.96	4.21	Zeta-carotene desaturase
*slr1254*	1.73	1.76	4.43	Phytoene dehydrogenase (phytoene desaturase)
*slr1877*	6.00	3.15	3.75	2-hydroxyhepta-2, 4-diene-1, 7-dioate isomerase
***Common stress response***				
*sll0248*	3.60	3.67	1.86	Flavodoxin
*sll1388*	3.21	1.58	1.82	Universal stress protein
*sll1988*	1.90	1.53	1.81	33 kDa chaperonin
*slr0093*	2.61	1.53	2.61	DnaJ protein, molecular chaperone
*slr1795*	2.43	1.71	2.16	Peptide methionine sulfoxide reductase
*slr1828*	3.38	3.33	11.00	Ferredoxin, petF-like protein
*slr1846*	1.53	2.25	4.71	Uncharacterized monothiol glutaredoxin
*slr1854*	4.09	1.59	4.38	General stress protein 18 (*gsp17* )
*slr2047*	2.06	1.65	1.62	Phosphate starvation-inducible protein
*ssl2250*	6.00	2.00	5.00	Bacterioferritin-associated ferredoxin
***Protein fate***				
*sll0616*	4.19	1.88	3.54	Preprotein translocase SecA subunit
*sll0716*	3.50	3.00	1.83	Probable signal peptidase I-1
*slr0835*	1.87	2.17	6.92	MoxR protein homolog
*slr0994*	2.22	1.62	2.30	Octanoyltransferase
*slr1046*	1.86	1.75	1.50	Putative TatA protein
*slr1204*	4.18	2.63	5.57	Putative serine protease HtrA
*slr1331*	2.94	2.26	3.98	Periplasmic processing protease
*ssr3307*	1.76	1.63	2.04	Preprotein translocase SecG subunit
***Regulatory functions***				
*sll0043*	3.20	3.20	2.21	Positive phototaxis histidine kinase
*sll0690*	5.00	1.57	6.00	Probable transcription regulator
*slr0640*	2.28	1.86	2.20	Two-component sensor histidine kinase
*slr0780*	1.84	1.70	2.33	Transcriptional repressor NrdR
*slr0947*	3.18	2.38	5.44	Response regulator for energy transfer from phycobilisomes to photosystems
*slr1037*	3.63	2.00	3.33	Two-component response regulator CheY subfamily
*slr1042*	3.60	2.45	1.72	Two-component response regulator CheY subfamily
*slr1414*	1.54	1.67	1.66	Two-component sensor histidine kinase
*slr1531*	3.00	1.83	1.55	Signal recognition particle protein
*slr1805*	1.53	3.81	4.66	Two-component sensor histidine kinase
*ssl0707*	2.67	1.93	2.78	Nitrogen regulatory protein P-II
***Storage compound biosynthesis***				
*slr1993*	8.75	1.76	4.79	PHA-specific beta-ketothiolase
*slr1994*	32.00	11.50	4.86	PHA-specific acetoacetyl-CoA reductase
*slr2002*	3.52	2.09	2.57	Cyanophycin synthetase
***Transport and binding proteins***				
*sll0374*	1.65	1.90	2.83	Urea transport system ATP-binding protein
*sll0689*	2.21	1.63	3.28	Na+/H + antiporter
*sll0759*	3.11	1.56	6.87	ABC transporter ATP-binding protein
*sll1041*	3.74	2.50	3.43	Similar to sulfate transport ATP-binding protein CysA
*sll1154*	1.76	2.80	3.98	NorA
*sll1164*	6.60	1.50	1.60	Uncharacterized transporter
*sll1428*	32.00	2.00	7.00	Probable sodium-dependent transporter
*sll1450*	2.89	3.98	5.27	Nitrate/nitrite transport system substrate-binding protein
*sll1451*	2.52	1.73	5.97	Nitrate/nitrite transport system permease protein
*sll1452*	1.98	1.66	3.12	Nitrate/nitrite transport system ATP-binding protein
*sll1481*	3.14	1.66	3.38	ABC-transporter membrane fusion protein
*sll1482*	3.30	1.51	2.57	ABC transporter permease protein
*sll1623*	2.15	1.56	1.91	ABC transporter ATP-binding protein
*slr1248*	2.57	2.63	3.00	Phosphate transport system permease protein PstC homolog
*slr1295*	2.33	1.79	5.92	Iron transport system substrate-binding protein
*slr1318*	1.50	2.50	1.77	Iron (III) dicitrate transport system ATP-binding protein
*slr1515*	2.56	1.61	3.47	Putative membrane protein required for bicarbonate uptake
*slr1729*	1.96	1.52	2.13	Potassium-transporting P-type ATPase B chain
*slr2131*	4.50	2.51	12.87	RND multidrug efflux transporter

**Table 3:** Comparison of ratios derived from RNA-seq and from RT-PCR analysis for selective genes

**Gene ID**	**Description**	**RT-PCR ratio**	**RNA-Seq ratio**
*sll0221*	Bacterioferritin comigratory protein	−1.18	−1.87
*sll0248*	Flavodoxin	1.31	3.67
*sll0629*	Photosystem I reaction center subunit PsaK 2	1.05	1.11
*sll1327*	ATP synthase gamma chain	1.29	1.10
*sll1734*	Hypothetical protein	1.52	1.29
*sll1796*	Cytochrome c6	1.74	2.38
*slr0288*	Glutamate--ammonia ligase	4.47	2.28
*slr0952*	Fructose-1,6-bisphosphatase class 1	−1.52	1.36
*slr1828*	Ferredoxin	1.96	3.33
*slr1909*	NarL subfamily response regulator	1.50	1.06

### Potential gene targets related to butanol tolerance

Our previous proteomic analysis found that the *Synechocystis* cells employed a combination of approaches to cope with butanol stress, and the responses included an induced common stress response, modifications of cell envelope, and induction of multiple transporters and signal transduction proteins against butanol stress [26]. Transcriptomic analysis showed very similar responses:

i. *Heat-shock and general stress proteins:* early analysis of butanol tolerance in both native and unnatural producing microorganisms showed that heat-shock proteins were relevant to tolerance [7,17]. Our quantitative proteomics found that DnaJ1 (Slr0093) was significantly induced at 48 h after butanol treatment [26]. At transcriptional level, we found that four genes involved in heat shock and general stress responses were induced (*i.e.*, *slr0093*, *sll1988*, *sll1388* and *slr1854*). In addition, *slr1204* encoding a putative serine protease (HtrA) and *slr0835* encoding a MoxR protein homolog were also up-regulated significantly by butanol (Table 2). HtrA-type serine proteases participate in folding and degradation of aberrant proteins and in processing and maturation of native proteins, and *htrA* mutation often conferred a pleiotropic phenotype that can include high sensitivity to various stress [28]. The MoxR family AAA+ proteins are ubiquitous proteins that employ the energy obtained from ATP hydrolysis to remodel proteins, DNA or RNA. Early studies have showed that some members of this protein group can potentially function as molecular chaperones involved in the assembly of protein complexes [29], and be involved in stress resistance and virulence in *Francisella tularensis*[30].

ii. *Oxidative stress response:* early studies showed that solvent like ethanol or butanol can challenge cells by causing increased production of highly reactive oxygen species (ROS) [31]. Transcriptomic analysis found that butanol induced expression of *slr1828* and *sll0248* genes encoding a *petF*-like ferredoxin and a flavodoxin protein (Table 2), respectively, consistent with the up-regulation of these two proteins in proteomics dataset. In addition, transcriptomic analysis showed that other genes involved in oxidative stress response, such as *ssl2250* encoding a bacterioferritin-associated ferredoxin, *slr1846* encoding a putative monothiol glutaredoxin and *slr1795* encoding a peptide methionine sulfoxide reductase were also up-regulated (Table 2). Recent study showed that bacterioferritin comigratory proteins, along with glutathione peroxidase-reductase, were responsible for detoxification of bentazone-derived peroxide in a *S. elongatus* PCC7942 mutant Mu2 [32]. Monothiol glutaredoxins was found with roles in actin cytoskeleton remodeling and cellular defenses against oxidative stress caused by ROS accumulation in *Saccharomyces cerevisiae* and *Schizosaccharomyces pombe*[33,34]. In addition, monothiol glutaredoxin (Slr1846) was found up-regulated by ethanol in *Synechocystis*[35]. Specific modifications of certain amino acid side chains are common during oxidative stress. Cysteine and methionine both contain a sulfur atom in their side chains and are among the most easily oxidized amino acids. Methionine sulfoxides can be reduced back to the methionines by peptide methionine sulfoxide reductase (MSR), providing cells with a mechanism to repair proteins damaged by reactive oxygen species rather than having them degraded and then re-synthesizing them *de novo*[36]. Induction of the methionine sulfoxide reductase by oxidative stress has been found in anaerobic *Desulfovibrio vulgaris*, *E. coli*, *S. cerevisiae* and *Synechocystis*[35-38].

iii. *Transporters:* transcriptomics analysis identified 19 membrane transporters were up-regulated. Among them only two genes, *sll0689* and *slr1512* which were in the same operon with butanol-induced *slr1515*, were identified in the previous proteomics analysis [26]. Interestingly, the up-regulated transporters involved a wide range of putative substrates, including iron, Na^+^/H^+^, nitrate/nitrite, phosphate, sodium, potassium, urea, bicarbonate and sulfate (Table 2). Moreover, many of these transporters were induced at significantly high fold changes, such as *slr2131* encoding a RND multidrug efflux transporter up-regulated 12.87 fold at 72 h, and *sll1428* encoding a probable sodium-dependent transporter up-regulated 32.0 folds. Other up-regulated genes included *sll1697* which encodes a well-studied multidrug efflux pump NorA [39]. Exact functions of these transporters in butanol tolerance may worth further investigation.

iv. *Protein translocation:* Bacteria have two major protein translocation systems, one of which is catalyzed by the Sec-dependent protein translocation system, and another is the Twin-arginine (Tat) protein translocation system [40,41]. Our proteomic analysis showed that SecE protein (Ssl3335) of Sec-dependent translocation system and Tha4 protein (Slr1047) of the Tat translocation system were up-regulated by butanol. Trnascriptomic analysis showed that *ssr3307* encoding a preprotein translocase SecG subunit, *sll0616* encoding a preprotein translocase SecA subunit and *slr1046* encoding a putative TatA protein, were up-regulated by butanol. Genes *slr1046* and *slr1047* were organized in the same operon. The results confirmed that enhanced protein translocation systems may be an important mechanism against butanol stress.

v. *Cell envelope:* Cell envelope is the important barrier in protecting cells. Consistent with proteomic results, our transcriptomic analysis also found that many genes involved in cell envelope function were up-regulated upon butanol exposure, such as *sll2010* encoding UDP-N-acetylmuramoylalanine--D-glutamate ligase, *slr0528* encoding UDP-N-acetylmuramoylalanyl-D-glutamate-2, 6-diaminopimelate ligase and *sll088* encoding UDP-N-acetylglucosamine--peptide n-acetylglucosaminyltransferase (Table 3). Their up-regulation was supposed to strengthen cell wall structure against butanol stress.

vi. *Regulatory genes:* Previous proteomic analysis showed that several signal transduction proteins involved in cell mobility (*i.e.* Che type) and nitrate induction, and repression of genes encoding nitrate respiration enzymes (*i.e.* NarL subfamily) were up-regulated by butanol [26]. Transcriptomics analysis identified 11 butanol-induced signal transduction genes. The induced genes included two Che type response regulators (*i.e. slr1042*, *slr1037*) and one putative phototaxis histidine kinase (*sll0043*) involved in cell mobility, and one gene (*ssl0707*) involved in nitrogen metabolism. Gene *ssl0707* encodes a nitrogen regulatory protein P-II belonging to the NtcA regulon in cyanobacteria [42]. Although the transcriptomic results confirmed that regulation of cell mobility and nitrogen responses are important in combating butanol stress, none of regulatory genes/proteins was identified in both transciptomic and proteomic datasets, suggesting the complicity of signal transduction in *Synechocystis*, and also the insufficiency to use any single ‘omics’ approach to characterize the complexity of biological systems [25]. To compare the proteomic and transcriptomic datasets quantitatively, 11 common genes/proteins up-regulated in both transcriptomics and proteomics datasets were listed in Table 4. The results also showed the very similar trends of up-regulation.

In our previous proteomic analysis, using a cutoff of 1.5-fold change and a *p*-value less than 0.05, we determined that 63 and 79 proteins were up-regulated between control and butanol treatments conditions at 24 h and 48 h, respectively; among which 35 proteins were up-regulated at both time points [26]. Comparison of proteomic and transcriptomic datasets showed that among the 278 genes up-regulated by butanol, 17 induced genes also had their corresponding proteins up-regulated (Table 4), 10 genes had their corresponding proteins down-regulated, and 251 induced genes have their protein levels unchanged. The finding that a relatively low number of genes and proteins shared the same up-regulation patterns, was probably due to the fact we used highly strict criteria in determining induced genes (*i.e.*, up-regulated at all three time points in this study). In spite of low correlation between the two datasets, the patterns of metabolic changes key to the butanol tolerance seemed similar, as described above for each of the functional categories.

**Table 4:** Quantitative comparison of transcriptomic and proteomic analyses *

**Gene ID**	**Transcriptomics analysis (fold change)**			**Proteomics analysis (fold change)**		**Description**
	**Butanol vs. Control at 24 h**	**Butanol *vs *. Control at 48 h**	**Butanol *vs. *Control at 72 h**	**Butanol *vs. *Control at 24 h**	**Butanol *vs. *Control at 48 h**	
*sll0135*	1.64	1.61	2.51		2.42	5′-methylthioadenosine phosphorylase
*slr1390*	1.83	1.62	2.74		1.52	Cell division protein FtsH
*sll1796*	3.70	2.38	9.75	3.80	2.91	Cytochrome C553
*slr0093*	2.61	1.53	2.61		1.93	DnaJ protein
*slr1330*	3.18	2.11	5.69	1.86		F0F1 ATP synthase subunit epsilon
*slr1828*	3.38	3.33	11.00	2.09	1.66	ferredoxin
*sll0248*	3.60	3.67	1.86	1.59	6.26	Flavodoxin FldA
*sll0335*	1.64	4.33	1.75		1.52	Hypothetical protein
*sll0470*	4.27	1.55	2.84		1.67	Hypothetical protein
*sll1618*	1.63	2.71	1.86	1.53	1.67	Hypothetical protein
*sll1895*	2.23	1.69	2.31		1.59	Hypothetical protein
*slr0643*	2.13	1.69	2.25	1.52	1.88	Hypothetical protein
*slr1046*	1.86	1.75	1.50	1.57		Hypothetical protein
*ssl0242*	2.63	2.53	2.47	1.79	1.66	Hypothetical protein
*ssl0352*	2.30	2.60	6.33	1.62	1.84	Hypothetical protein
*sll0689*	2.21	1.63	3.28	1.70	1.93	Na/H + antiporter
*slr1755*	1.97	1.85	3.44	1.56		NAD(P)H-dependent glycerol-3-phosphate dehydrogenase

* Proteomics data from Tian et al., (2012), in which only proteomes at 24 and 48 h were measured. The fold changes of proteomics data are average of replicates.

One goal of the integrated OMICS analysis is to achieve a complete coverage of cellular molecules by using complementary techniques targeting different levels of information (*i.e.*, RNA, protein or metabolites) [25]. In this study, our transcriptomic analysis also revealed new cellular responses which were not observed in the previous proteomic analysis [26]: *i*) *Enhanced production of storage compounds*: Polyhydroxyalkanoates (PHAs) are common carbon storage compounds that are accumulated during unbalanced growth conditions [43]. Two genes involved in PHA biosynthesis, *slr1994* encoding a PHA-specific acetoacetyl-CoA reductase and *slr1993* encoding a PHA-specific beta-ketothiolase were found up-regulated by butanol (Table 2). Cyanophycin is a non-ribosomally synthesized peptide, composed of arginine and aspartic acid, accumulates when cells are grown under all unbalanced nutrient conditions except nitrogen starvation, and has been considered as a primary nitrogen reserve compound in cyanobacteria [44]. Transcriptomic analysis showed that the key gene involved in cyanophycin synthesis, *slr2002* encoding cyanophycin synthetase was up-regulated by butanol (Table 2). Although PHA and cyanophycin accumulation has been reported for many natural stress conditions, it may worth further investigation how these pathways respond to butanol stress; *ii*) *Enhanced carotenoid biosynthesis*: three genes involved in carotenoid biosynthesis were up-regulated: *slr1254* encoding phytoene desaturase, *slr0940* encoding zeta-carotene desaturase and *slr0899* encoding cyanate lyase (Table 2). The results were consistent with the increased photosynthetic activity of *Synechocystis* upon butanol stress [26]. Carotenoid biosynthesis has been found up-regulated by strong light in *Synechococcus* PCC7942 [45], and in stress-tolerant mutants of *Haematococcus pluvialis*[46]. The results provided further evidences that the integrated OMICS approach could be advantageous in revealing global cellular responses.

### Metabolomic signatures related to butanol response

GC-MS based metabolomic analysis was used to characterize the time-series metabolic responses of *Synechocystis* to butanol exposure, with unperturbed cultures as controls. Cell samples used for metabolomic analysis were collected at 24, 48 and 72 h, respectively, the identical time points of sampling for transcriptomic analysis. Three biological replicates were collected for each time point and treatment, thereby yielding a total of 18 samples. The analysis showed that a total of 73 metabolites were chemically identified with great confidence. Although more metabolites were detected in butanol-treated samples (70.4 ± 2.74) than the control samples (64.12 ± 4.01), the number of metabolites identified varied only slightly within control or treatment bins, implying an overall good analytical quality. To further assess the reproducibility of GC-MS metabolomics, we analyzed three technical replicates of one selected sample, and the results showed that most of the metabolites were identified in technical replicates (Date not shown).

**Figure 2:**
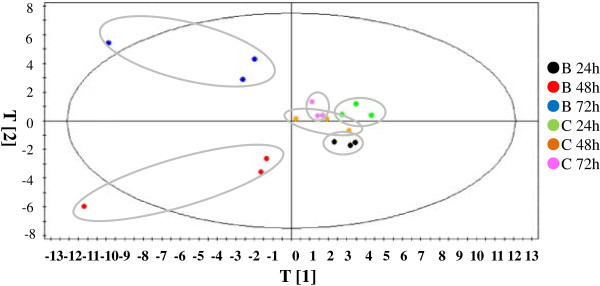
**PCA score plot of metabolomic profiles of *Synechocystis *along the treatment time course.** Samples with or without butanol treatments were indicated by different colors. The biological replicates were circled for the same conditions.

The score plot of principal component analysis (PCA) was applied to evaluate the similarities and differences between the 18 metabolomic profiles (Figure 2). The score plot revealed the following features: *i*) the samples with or without butanol treatment at different time points were distinctly separated, suggesting significant metabolic differences between samples; *ii*) for the control samples, metabolic changes along the time courses were relatively small, as showed by the clustering patterns of 9 samples; and *iii*) when compared with controls, significant metabolic changes were observed for butanol-treated samples, especially for samples with 48 and 72 h butanol treatments. One of the butanol-treated biological replicates was slightly different from other two biological replicates at 48 h and 72 h, probably due to the fact the long-term butanol treatment has caused significant cell aggregation [26], which increased the sample heterogeneity. Nevertheless, the overall similar response patterns can still be observed in these replicate samples according to their position in the score plot (Figure 2). Using a cutoff ratio of 1.5 fold between butanol-treated and control samples, and change in at least 5 out of 9 replicate ratios in any time point, we determined 46 metabolites were differentially regulated, in which 35, 41 and 38 metabolites were detected in 24, 48 and 72 h, respectively (Table 5). Pattern analysis showed the 48 metabolites can be divided into at least 6 clusters according to their changes along the treatment time courses. For example, Cluster I included 7 metabolites up-regulated in all three time points, while Cluster II included 7 metabolites up-regulated only in 48 and 72 h after butanol exposure (Table 5).

**Table 5:** Differentially regulated metabolites *

**Metabolites**	**24 h**	**48 h**	**72 h**
***Cluster I***			
Aspartic acid	4	9	9
Glycerol 1-phosphate	7	9	6
3-phosphoglycerate	3	9	3
Citric acid	2	9	6
D-allose	9	3	9
Adenosine	5	9	8
Urea	1	7	3
***Cluster II***			
Oxalic acid	0	9	6
Glycine	0	4	5
Succinic acid	0	6	6
L-glutamic acid (dehydrated)	0	9	6
Isocitric acid	0	6	6
Myristic acid	0	6	8
Sucrose	0	6	9
***Cluster III***			
1,3 Propanediol	6	−6	−6
L-alanine	3	−3	−9
Itaconic acid	9	−4	−9
Spermidine	4	−9	−5
***Cluster IV***			
Methyl palmitate	9	−5	6
Talose	9	−4	9
Benzene-1,2,4-triol	2	−3	6
Adrenaline	7	−6	9
Lauric acid	9	−8	7
***Cluster V***			
L-threonine	7	9	0
D-malic acid	5	3	0
L-serine	6	5	0
***Cluster VI***			
L-pyroglutamic acid	−3	−9	−3
Linoleic acid	7	0	9
L-(+) lactic acid	2	−6	0
Pyruvic acid	−6	0	9
3-hydroxypyridine	0	−5	9
4-hydroxypyridine	0	−3	8
Malonic acid	−5	−3	8
Caprylic acid	−6	0	9
Octane	−2	−5	8
Glycerol	6	−5	0
Glyceric acid	8	−9	0
Uracil	−8	6	4
Putrescine	−8	0	2
Tagatose	−5	0	9
Palmitic acid	0	−6	5
Methyl stearate	−9	9	9
D-glucose-6-phosphate	−3	8	9
Stearic acid	0	−8	5
Arachidic acid	−3	−8	5
D-(+) trehalose	−3	9	0

* For each time point, three biological replicates of butanol-treated samples were compared with three biological replicates of control, generating 9 ratios. For each ratio, ratio > 1.5 was assigned as “ + 1”, ratio< − 1.5 as “-1”, and −1.5 < ratio < 1.5 as “0”. The sums of the nine ratios for each metabolite at any time point were provided.

Metabolomic analysis has identified several metabolites induced by butanol treatment, including 3-phosphoglycerate (3-PG) and glycerol 1-phosphate induced significantly in all three time points, serine induced at 24 and 48 h, and glycine induced at 48 and 72 h after butanol exposure, respectively. The findings were consistent with early studies which showed 3-phosphoglycerate is increasingly withdrawn from the Calvin cycle in *S. elongatus* PCC 7942 under iron limitation stress [47]. In addition, phosphoglycerate kinase that catalyzes the production of 3-phosphoglycerate from 1,3-bisphosphoglycerate was also found induced in *Anabaena* sp. PCC7120 under arsenic stress [48]. Moreover, early study has shown that the intracellular levels of organic acids (glyceric, glycolic and glyoxylic acids) and amino acids (glycine and serine) were elevated in salt-treated *Anabaena* sp. PCC 7120 as compared to those in the control cells [49]. The results suggested that these metabolites could be important part of metabolic responses to both butanol and general environmental stresses.

Previous proteomic study found that a common stress response of *Synechocystis* under various environmental perturbations, irrespective of amplitude and duration, is the activation of atypical pathways for the acquisition of carbon and nitrogen from urea and arginine, as evidenced by the significant up-regulation of urease that converts urea into CO_2_ and ammonia, under most conditions [50]. Our metabolomic analysis showed that urea was induced by butanol, especially at 48 and 72 h. Previous proteomic analysis showed that cyanophycinase, involved in the breakdown of cyanophycin, a storage molecule for excess carbon and nitrogen, into arginine and aspartic acid, was moderately up-regulated under several conditions [50]. Arginine and aspartic acid can be further converted to glutamate and succinate, respectively [51]. Metabolomic analysis showed that aspartic acid was significantly induced at all three time points, and succinic acid and L-glutamic acid were both induced at 48 and 72 h by butanol treatment. These results implied that a similar up-regulated degradation of cyanophycin may also occur under butanol stress.

Integrated transcriptomic and metabolomic analysis has been proposed as a powerful tool to build the relationship between information elements (*i.e.*, genes/transcripts) and functional elements (*i.e.*, metabolites) in cells [25,52,53]. In one recent study, integrated transcriptomic and metabolomic approach was used to determine the infection mechanism of *Rhodococcus fascians* into *Arabidopsis thaliana*. The transcriptomic analysis showed a significant impact of infection on the primary metabolism of the host, which was then confirmed by subsequent metabolite analysis, for example, invertase transcripts and activities strongly enhanced upon infection, may related to the increase in the hexose:sucrose ratio [54]. In another study to compare the aerobic and anaerobic fermentations of *Zymomonas mobilis*, researchers found that greater amounts of end products such as acetate, lactate and acetoin were detected under aerobic conditions, while no change in terms of gene expression was found between aerobic and anaerobic conditions in the early exponential growth phase [55], implying the importance to applying integrated technology in uncovering related molecular mechanism. In this study, although only small number of metabolites can be chemically classified in *Synechocystis*, the metabolomic analysis found increased abundances of aspartic acid and serine, which was consistent with the induction of *slr0550* encoding dihydrodipicolinate synthase involved in aspartate pathway, and *sll0455* encoding homoserine dehydrogenase involved in serine pathway, respectively (Table 5). In addition, increased abundance of glutamic acid inside the cells was correlated with up-regulation of *sll1883* encoding bifunctional ornithine acetyltransferase/N-acetylglutamate synthase protein, *sll0461* encoding gamma-glutamyl phosphate reductase, *slr0288* encoding glutamate--ammonia ligase, and *slr1898* encoding acetylglutamate kinase that are involved in metabolism of glutamate family amino acids (Table 5). Moreover, metabolomic analysis showed the increased abundances of intermediates in the glycolysis pathway, such as glucose-6-P and 3-PG, consistent with the induction of two key genes, *slr0752* encoding phosphopyruvate hydratase and *sll0745* encoding 6-phosphofructokinase in the glycolysis pathway. Consistent with this result, up-regulation of glycolysis has been reported for various microbes under stress condition [56,57]. In a recent ^13^C-based flux analysis, a thermophilic ethanol-tolerant *Geobacillus thermoglucosidasius* M10EXG was found to prefer glycolysis, the pentose phosphate pathway and the TCA cycle for glucose metabolism [58]. On the other hand, for some of differentially regulated metabolites identified, such as urea and cyanophycin, no change was observed for their functionally-related genes in the transcriptomic datasets, which may be due to multiple factors, such as the snapshot nature of the analysis and the different stability of RNA molecules [25]. Nevertheless, the results further demonstrated that transcriptomic and metabolomic technologies could be complementary to each other, allowing better decipherment of cellular responses of *Synechocystis* under butanol stress.

### Validation of potential tolerance targets

**Figure 3:**
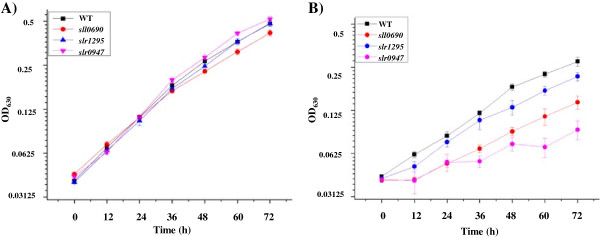
**Comparative analysis of butanol tolerance of wild-type strain and mutants. A)** Growth time courses of wild type, *Δsll0690*, *Δslr0947* and *Δslr1295* mutants in normal BG11 medium; **B)** Growth time courses of wild type, *Δsll0690* mutant, *Δslr0947* mutant and *Δslr1295* mutant in BG11 media supplemented with 0.25% (*v*/*v*) butanol.

Three genes, *sll0690*, *slr0947* and *slr1295* which were found induced by butanol exposure at all three time points (*i.e.* 24, 48 and 72 h) (Table 2), were selected for construction of knockout mutants and for validation of their involvement in butanol resistance. *sll0690* encoding a probable transcription regulator, was up-regulated 5–6 folds, *slr0947* encoding an OmpR-type DNA-binding response regulator, was up-regulated 2.4-5.5 folds, and *slr1295* encoding an iron transport system substrate-binding protein was up-regulated 1.8-5.9 folds by butanol, respectively. Two corresponding proteins of the genes, Slr0947 and Slr1295, were identified in our previous proteomic analysis, in which they were also slightly up-regulated 1.16-1.55 and 1.16-1.57 folds after butanol treatment for 48 h, respectively [26]. After confirmed by PCR and sequencing, the mutants were grown in parallel with wild type *Synechocystis* in both normal BG11 medium and the BG11 medium supplemented with 0.25% (*v*/*v*) butanol. Comparative analysis showed that although there is no visible difference in terms of growth patterns between the wild type and all three mutants in the regular BG11 medium (Figure 3A), gene disruption of *sll0690*, *slr0947* and *slr1295* led to increased butanol sensitivity, suggesting they were involved in butanol resistance (Figure 3B). Currently little is known how these genes are involved in butanol tolerance, although early studies have found that the *slr1295* gene product, a periplasm-located component of an iron transporter, has a function in protecting photosystem (PS) II [59] and was induced under salt-stress condition [60]; and the *slr0947* gene was involved in the regulation of the coupling of phycobilisomes to photosynthetic reaction centers, and reduction of the copy number of *slr0947* resulted in decreased efficiency of energy transfer from phycobilisomes to photosystem II relative to photosystem I [61].

## Conclusions

RNA-Seq based transcriptomics coupled with RT-PCR and GC-MS metabolomics were used to determine gene targets related to butanol tolerance in *Synechocystis*. Although the overall cellular responses revealed by transcriptomics and metabolomics were very similar to those revealed by our previous proteomic analysis, the genes/proteins involved in each type of responses were not always identical, consistent with recent conclusions that only a weak correlation exists between large-scale transcriptomic and proteomic datasets so that an integrative analysis of multiple levels of gene expression would be necessary and valuable [62]. A comprehensive transcriptomic and metabolomic analysis with proteomic analysis led to identification of putative gene targets which may be involved in butanol tolerance. By constructing KO mutants and analyzing their butanol resistance, we validated three potential gene targets identified by the integrated OMICS approaches. In the future, once further functional characterization of these candidate genes completed, it is possible they can serve as target genes to engineer more robust butanol-tolerant cyanobacterial hosts.

## Materials and methods

### Bacterial growth conditions and butanol treatment

*Synechocystis* sp. PCC 6803 was grown in BG11 medium (pH 7.5) as described previously [26,27]. Butanol of 0.20% (*v*/*v*) was added at the beginning of cultivation. Cells were collected by centrifugation at 8,000 × *g* for 10 min at 4°C.

### RNA preparation and cDNA synthesis

Approximately 10 mg of cell pellets were frozen by liquid nitrogen immediately after centrifugation and cell walls were broken with mechanical cracking at low temperature. Cell pellets were then resuspended in Trizol reagent (Ambion, Austin, TX) and mixed well by vortex. Total RNA extraction was achieved using a miRNeasy Mini Kit (Qiagen, Valencia, CA). Contaminating DNA in RNA samples was removed with DNase I according to the instruction in the miRNeasy Mini Manual (Qiagen, Valencia, CA). The RNA quality and quantity were determined using Agilent 2100 Bioanalyzer (Agilent, Santa Clara, CA) and subjected to cDNA synthesis. The RNA integrity number (RIN) of every RNA sample used for sequencing was more than 8.0. For each sample, 500 ng total RNA were subjected to cDNA synthesis using a NuGEN Ovation® Prokaryotic RNA-Seq System according to manufacturer’s protocol (NuGEN, San Carlos, CA). The resulting double-stranded cDNA was purified using the MinElute Reaction Cleanup Kit (Qiagen, Valencia, CA).

### RNA-seq library preparation

The double-stranded cDNA obtained was subjected to library preparation using the Illumina TruSeqTM RNA Sample Preparation Kit (Illumina, San Diego, CA), through a four-step protocol of end repairing, adenylate 3’ ends adding, adapter ligation, and cDNA template enrichment. To determine the quality of libraries, a Qubit® 2.0 Fluorometer and Qubit™ dsDNA HS (Invitrogen, Grand Island, NY) were first used to determine the DNA concentration of the libraries, and then FlashGel DNA Cassette (Lonza, USA) or Agilent Technologies 2100 Bioanalyzer (Agilent, Santa Clara, CA) was used to determine the product size of the libraries, with good libraries typically around 300 bp.

### Next-generation sequencing

RNA 2 × 100 bp paired-end sequencing was performed using Illumina’s Solexa Genome Analyzer II using the standard protocol. The cDNA library of each sample was loaded to a single lane of an Illumina flow cell. The image deconvolution and calculation of quality value were performed using Goat module (Firecrest *v*.1.4.0 and Bustard *v*.1.4.0 programs) of Illumina pipeline *v*.1.4.

### Transcriptomics data analysis

Sequence reads were pre-processed using FASTX Toolkit (*v*. 0.0.13) to remove low-quality bases, and reads shorter than 20 bp. The qualified sequence reads were then mapped to non-coding RNA (*nc*RNA) sequences using Bowtie (*v*. 2.0.0) with default settings. Genome sequences (including *nc*RNA sequences) and annotation information of *Synechocystis* sp. PCC 6803 were downloaded from NCBI (Downloaded on April 22, 2012) [27]. Reads that mapped to *nc*RNA sequences were excluded from further analysis in this study. For paired-end Illumina reads, both pairs were removed if either pair mapped to rRNA. Remaining reads were mapped to the *Synechocystis* genome using Bowtie (*v*. 2.0.0) with the default parameters. For gene expression determination, we performed a standard calculation of Reads Per Kilobase of Gene per Million Mapped Reads (RPKM) [63]. We performed comparative transcriptome analysis for all three time points (*i.e.*, 24, 48 and 72 h). To identify the reliable gene targets related to butanol tolerance, only the genes with 1.5-fold induction by butanol at all three time points were regarded as up-regulated genes.

### Quantitative real-time RT-PCR analysis

The identical RNA samples used for transcriptomics analysis as described above were used for RT-qPCR analysis. cDNAs were synthesized using RevertAidTM Reverse Transcriptase (Fermentas, Glen Burnie, MD). The qPCR reaction was carried out in 20 μl reactions containing 10 μl of SYBR® Green PCR Master Mix (Applied Biosystems, Foster City, CA), and 2 μl of each PCR primer at 2 mM, employing the StepOnePlus™ Real-Time PCR System (Applied Biosystems, Foster City, CA), under the following condition: 50°C for 2 min and 95°C for 10 min, followed by 40 cycles of 95°C for 15 s and 60°C for 1 min. Quantification of gene expression was determined according to standard process of RT-PCR which used serial dilutions of known concentration of chromosome DNA as template to make a standard curve. The *rnpB* gene (*6803 s01*) encoding RNase P subunit B was used as an internal control according to the previous publication [64]. Three technical replicates were performed for each gene. Data analysis was carried out using the StepOnePlus analytical software (Applied Biosystems, Foster City, CA). Data was presented as ratios of the amount of normalized transcript in the treatment to that from the control. The gene ID and their related primer sequences used for real-time RT-PCR analysis were listed in Additional file 1: Table S1.

### GC-MS based metabolomics analysis

All chemicals used for metabolome isolation and GC/MS analysis were obtained from Sigma-Aldrich (Taufkirchen, Germany). Cells were collected from control and butanol-treated (0.2% *v*/*v*) cultures at 24, 48 and 72 h, respectively. Three biological replicates were established for each sample, and every sample was analyzed three times. For each sample, cells from 5 to 20 mL culture, equivalent to 10^8^ cells mL^-1^, were collected by centrifugation at 8000 × *g* for 10 min at 4°C (Eppendorf 5430R, Hamburg, Germany). The cell pellets were frozen in liquid nitrogen and then stored at −80°C before use. *i*) Metabolome extraction: cells were re-suspended in 1 mL cold 10:3:1 (*v*/*v*/*v*) methanol: chloroform: H_2_O solution (MCW), and frozen in liquid nitrogen and thawed for five times. Supernatants were collected by centrifugation at 14,000 × *g* for 3 min at 4°C (Eppendorf 5430R, Hamburg, Germany). To normalize variations across samples, an internal standard (IS) solution (100 μg/mL U- 13C-sorbitol, 5 μL) was added to 100 μL supernatant in a 1.5-mL microtube before it was dried by vacuum centrifugation for 2–3 h (4°C). *ii*) Sample derivatization: derivatization was conducted according to the two-stage technique by Roessner *et al*. (2001) [65]. The samples were dissolved in 10 μL methoxyamine hydrochloride (40 mg/mL in pyridine) and shaken at 30°C for 90 min, then were added with 90 μL N-methyl- N -(trimethylsilyl) trifluoroacetamide (MSTFA) and incubated at 37°C for 30 min to trimethylsilylate the polar functional groups. The derivate samples were collected by centrifugation at 14,000 × *g* for 3 min before GC/MS analysis. *iii*) GC-MS analysis: sample analysis was performed on a GC-MS system-GC 7890 coupled to an MSD 5975 (Agilent Technologies, Inc., Santa Clara, CA, USA) equipped with a HP-5MS capillary column (30 m × 250 mm id). 2 μL derivatized sample was injected in splitless mode at 230°C injector temperature. The GC was operated at constant flow of 1 mL/min helium. The temperature program started isocratic at 45°C for 2 min, followed by temperature ramping of 5°C/ min to a final temperature of 280°C, and then held constant for additional 2 min. The range of mass scan was m/z 38–650. *iv*) Data processing and statistical analysis: The mass fragmentation spectrum was analyzed using the Automated Mass Spectral Deconvolution and Identification System (AMDIS) [66] to identify the compounds by matching the data with Fiehn Library [67] and the mass spectral library of the National Institute of Standards and Technology (NIST). Peak areas of all identified metabolites were normalized against the internal standard and the acquired relative abundances for each identified metabolite were used for future data analysis.

All metabolomic profile data was first normalized by internal control and cell numbers, and then subjected to Principal Component Analysis using software SIMCA-P 11.5 [68]. Differentially regulated metabolites were determined using a threshold of fold change greater than 1.5 between butanol-treated samples and controls. For each time point, three biological replicates of butanol-treated samples were compared with three biological replicates of control, generating 9 ratios. For each ratio, *r* > 1.5 was assigned as “ + 1”, *r* < −1.5 as “-1”, and −1.5 < *r* < 1.5 as “0”. The sums of the nine ratios for each metabolite at any time point were provided in Table 5.

### Construction and analysis of knockout mutants

A fusion PCR based method was employed for the construction of gene knockout fragments [69]. Briefly, for the gene target selected, three sets of primers were designed to amplify a linear DNA fragment containing the chloramphenicol resistance cassette (amplified from a plasmid pACYC184) with two flanking arms of DNA upstream and downstream of the targeted gene. The linear fused PCR amplicon was used directly for transformation into *Synechocystis* by natural transformation. The chloramphenicol-resistant transformants were obtained and passed several times on fresh BG11 plates supplemented with 10 μg/ml chloramphenicol to achieve complete chromosome segregation. Three genes, *sll0690*, *slr0947* and *slr1295* that have been found differentially regulated by butanol exposure, were selected for construction of gene knockout mutants. PCR primers for mutant construction and validation were listed in Additional file 1: Table S1. Full segregation for *sll0690* and *slr1295* genes was confirmed by PCR. For Δ*slr0947* mutant, we found that it contained trace amount of original wild-type band in the DNA gels even after more than ten passages, it may worth further investigation whether *slr0947* is a lethal gene for the condition. Comparative growth analysis of the wild type 6803 and the mutants were performed in 100-mL flasks each with 10 mL BG11 medium with or without 0.25% (*v*/*v*) butanol. Cultivation conditions are the same as described above. Growth analysis was performed in biological triplicates.

## Abbreviations

GC-MS: Gas chromatography–Mass spectrometry; Glucose-6-P: glucose-6-phosphate; iTRAQ: Isobaric tag for relative and absolute quantitation; LC-MS/MS: Liquid chromatography-tandem mass spectrometry; ncRNA: non-coding RNA; PCA: Principal component analysis; 3-PG: 3-phosphoglycerate; RPKM: Reads per kilobase of gene per million mapped reads; ROS: Reactive oxygen species; RT-PCR: Reverse-transcript PCR.

## Competing interests

The authors declare no competing interests.

## Authors’ contributions

XR, ZS, LC and JW carried out cultivation and transcriptomics analysis. LC, XR, ZS and JL carried out the RT-PCR, mutant construction and phenotypic analysis. HZ, LC, JW, JQ and WZ finished the statistical analysis for transcripomic data. MS, XT, LC and WZ carried out the metabolomics experiments and analysis. LC, JW and WZ conceived of the study, participated in its design and coordination. HZ, JW, LC and WZ drafted the manuscript. All authors read and approved the final manuscript.

## Supplementary Material

Additional file 1: Table S1 Primers for RT-PCR analysis and mutant construction. Click here for file
